# Deep learning–assisted 3D CT angiography for supply-vessel localization in inflammatory peripheral pulmonary artery pseudoaneurysms

**DOI:** 10.3389/fmed.2026.1810868

**Published:** 2026-07-07

**Authors:** Lianjing Li, Chuzhao Li, Neng Wang, Xiaodie Xu, Dexin Liu, Chaohui Lin, Youcao Lin, Yunfeng Chen

**Affiliations:** 1 Department of Pulmonary Medicine, Nanan Haidu Hospital, Affiliated Hospital of the Integrated Healthcare System, The Second Affiliated Hospital of Fujian Medical University, Nanan, Fujian, China; 2 Department of Pulmonary Medicine, Fujian Medical University Union Hospital, Fuzhou, Fujian, China; 3 Fujian Key Laboratory of Lung Stem Cells, Key Laboratory of Sleep Medicine, Department of Pulmonary and Critical Care Medicine, Fujian Provincial Clinical Research Center of Interventional Respirology, The Second Affiliated Hospital of Fujian Medical University, Quanzhou, Fujian, China

**Keywords:** 3D reconstruction, CT angiography, deep learning, deviation analysis, digital subtraction angiography, inflammatory PAP, Pulmonary artery pseudoaneurysm, V-Net

## Abstract

Inflammatory peripheral pulmonary artery pseudoaneurysms (PAP) are rare but potentially fatal causes of massive hemoptysis. Identifying the true parent/supply vessel before intervention is challenging, particularly when bronchial-pulmonary (B–P) shunts and dual systemic pulmonary supply is present. Digital subtraction angiography (DSA) is often invasive and exploratory. We developed a deep learning–assisted 3D-CT angiography (3D-CTA) workflow for localizing PAP supply vessels and compared its findings to DSA. The workflow includes V-Net–based segmentation of the PAP and pulmonary arterial tree from chest CTA, followed by 3D reconstruction, interactive vessel tracing, and geometry verification. Retrospective evaluation in six patients with inflammatory peripheral PAP (angiographic subtypes A–C) confirmed by CTA and DSA showed that all six datasets were successfully reconstructed into 3D models. Segmentation achieved a mean Dice similarity coefficient (DSC) of 0.884 for PAP and 0.932 for the PA tree. Supply-vessel localization was fully concordant with DSA in four cases and partially concordant in two cases. Given the substantial procedural burden of DSA (mean duration 212 min), the proposed 3D-CTA roadmap can streamline endovascular planning and potentially reduce the need for extensive DSA procedures. This pilot study provides preliminary evidence supporting the feasibility of a deep learning–assisted 3D-CTA workflow for PAP supply-vessel localization. Given the small sample size, these findings should be interpreted cautiously, and further validation in larger cohorts is required before clinical generalization.

## 1 Introduction

Massive hemoptysis is a medical emergency that can rapidly lead to airway compromise and hemodynamic instability (1). Clinically, it is commonly defined as a hemoptysis volume ≥500 mL within 24 h or >100 mL in a single episode, and smaller volumes may still qualify if accompanied by respiratory or circulatory failure (2). Management typically requires multidisciplinary coordination, including airway protection, bronchoscopic localization, and definitive hemostasis procedures (3). Bronchial artery embolization (BAE) is widely used as a first-line option because it is less invasive and provides a high immediate hemostasis rate. However, persistent or recurrent bleeding after BAE raises concern that the bleeding source may involve the pulmonary arterial system rather than the systemic bronchial circulation (4). Pulmonary artery pseudoaneurysm (PAP) is a rare but high-risk vascular lesion that can arise when infection, trauma, or iatrogenic injury disrupts the pulmonary arterial wall, leading to a contained rupture surrounded by fibrous tissue rather than a normal three-layer arterial wall (5). Because pseudoaneurysms lack normal wall integrity, they may rupture more easily and precipitate life-threatening hemorrhage (6). Importantly, the lung has dual vascular supplies, and PAP blood supply may originate from systemic circulation, pulmonary circulation, or both; therefore, embolization targeting only the bronchial arteries may be insufficient in selected cases (7). In clinical practice, this complexity is further amplified by bronchial–pulmonary (B–P) shunts and multi-branch involvement, where the true parent/supply vessel is not readily apparent from conventional imaging (8). Chest CTA is widely regarded as a key diagnostic tool for PAP because it can depict characteristic vascular outpouchings and associated arterial changes with high spatial detail (9). Nevertheless, translating 2D CTA findings into a reliable, procedure-ready understanding of which branch to catheterize first remains challenging, especially when pulmonary arterial branches are numerous and selective angiography cannot practically be performed “branch-by-branch” during intervention (10). As a result, DSA may become an exploratory and time-consuming step. In our pilot cohort, DSA imposed substantial procedural burden, with a mean duration of 212 min, a type C mean duration of 360.3 min, and a longest case reaching 6 h, with extensive iodinated contrast consumption. These observations highlight an unmet need for a non-invasive, pre-procedural approach that can narrow the angiographic search space and improve confidence in supply-vessel selection.

Automated 3D modeling offers a potential solution by converting CTA volumes into patient-specific vascular representations that clinicians can interrogate interactively. Recent work in small-lesion CTA segmentation has demonstrated that 3D convolutional neural networks (3D CNNs) can achieve meaningful segmentation performance under small-sample constraints, and that preprocessing choices such as patch size can significantly influence performance, e.g., a 48 × 48 × 48 patch configuration yielded higher DSC and lower HD than smaller or larger patches in intracranial aneurysm segmentation experiments. Beyond overlap metrics, that study further emphasized evaluating geometric fidelity through 3D model deviation analysis using maximum distance, average distance, standard deviation, and RMS to quantify shape bias that may matter for downstream modeling and procedural planning. These methodological insights are directly relevant to inflammatory peripheral PAP because the clinical utility hinges not only on detecting the lesion but also on accurately representing its spatial relationship to nearby branches for parent-vessel tracing. In parallel, translational research on pseudoaneurysm modeling has shown that when CT-based segmentation yields reliable geometry, the digital model can be extended to procedure rehearsal and training through 3D printing, with accuracy assessed by Bland–Altman agreement of diameters/angles at predefined anatomical locations and feasibility supported by simulated DSA and coil embolization on printed models. While inflammatory peripheral PAP differs anatomically from peripheral femoral pseudoaneurysm, these validation concepts provide a structured pathway for future “digital-to-physical” extension once a robust 3D-CTA workflow is established.

Recent progress in thoracic CT segmentation has produced several improved deep-learning frameworks for lung-region and lung-nodule analysis. Delfan et al. proposed CT-LungNet, which combines a 2.5-dimensional CT representation with an InceptionV3-based U-Net to improve generalizability for lung tissue segmentation across multiple thoracic CT datasets (11). In parallel, recent V-Net-based nodule-segmentation studies have introduced attention mechanisms, threshold-aware feature separation, selective-kernel modules, multiscale feature extraction, coordinate attention, and edge-enhancement strategies to address small lesion size, irregular nodule morphology, fuzzy boundaries, and limited annotated data (12–14). These studies demonstrate that modern pulmonary CT segmentation has moved beyond standard encoder–decoder designs toward more targeted architectures that preserve small-region features and improve robustness in complex lung environments.

However, most existing lung-region and lung-nodule segmentation studies are primarily designed for parenchymal lung extraction, nodule delineation, or detection/classification support. In contrast, inflammatory peripheral pulmonary artery pseudoaneurysm (PAP) presents a different clinical and computational problem: the target is not only a small lesion but also a vascular abnormality whose clinical relevance depends on its three-dimensional relationship with adjacent pulmonary arterial branches and possible systemic–pulmonary supply pathways. Therefore, accurate segmentation alone is insufficient; the reconstruction must preserve the sac–neck–branch relationship and support branch-level parent/supply-vessel tracing before DSA. To address this gap, the present workflow uses an improved V-Net tailored for small vascular targets, anisotropic CTA data, derivative-enhanced boundary representation, geometry-preserving 3D reconstruction, and DSA-based interventional cross-validation. Recent engineering-oriented machine-learning studies have similarly emphasized task-specific model design, interpretability, and structural fidelity in complex systems (15–18); consistent with this direction, our work prioritizes clinically actionable vascular roadmap generation rather than generic lesion segmentation alone.

On this basis, we propose a deep learning–assisted 3D-CTA workflow for inflammatory peripheral PAP that aims to bridge the gap between diagnostic CTA and interventional decision-making. Our approach integrates: (i) improved V-Net–based segmentation and structural extraction to obtain the pulmonary arterial tree and PAP target (19), (ii) 3D reconstruction and interactive rendering that allow clinicians to visualize the PAP and trace candidate supply vessels (20), and (iii) geometric verification using 3D deviation analysis alongside pragmatic interventional cross-validation against DSA (21). The workflow is evaluated retrospectively in six inflammatory peripheral PAP cases confirmed by chest CTA and DSA, spanning angiographic subtypes A–C. We hypothesize that a segmentation-driven, geometry-aware 3D-CTA representation can improve the pre-procedural localization of supply vessels and support more targeted angiographic exploration, thereby reducing the procedural burden associated with exploratory DSA in complex PAP presentations. This pilot study presents three key contributions: (1) a clinically grounded deep learning–assisted 3D-CTA workflow specifically designed for supply-vessel localization in inflammatory peripheral pulmonary artery pseudoaneurysms (PAP), moving beyond mere lesion detection to functional procedural planning; (2) integration of 3D deviation analysis to ensure geometric fidelity, complementing traditional overlap metrics; and (3) practical interventional cross-validation using digital subtraction angiography (DSA) as the reference standard within a real-world clinical cohort. Given the limited sample size, this study is designed as a pilot feasibility investigation rather than a definitive validation of clinical performance. Unlike approaches that rely on transfer learning or hybrid 2.5D representations, our method focuses on a fully 3D convolutional framework tailored for vascular structure segmentation. Specifically, the proposed improved V-Net architecture emphasizes anisotropic convolution and geometry-aware feature preservation, which are particularly suited for capturing small, elongated vascular structures such as PAP and supply vessels. While techniques such as attention mechanisms and multi-scale fusion have demonstrated promising results in related tasks, their integration into vascular-focused models remains an area for future investigation.

## 2 Materials and method

### 2.1 Study design, ethics, and reporting

This retrospective pilot study was reviewed and approved by the Ethics Committee of the Second Affiliated Hospital of Fujian Medical University (Approval No. 2022-350) and conducted as a HIPAA-compliant study with waiver of informed consent (22). The study was designed to evaluate a deep learning–assisted 3D-CTA workflow for supply-vessel localization in inflammatory peripheral pulmonary artery pseudoaneurysms (PAP), with interventional cross-validation using digital subtraction angiography (DSA). Due to the rarity of inflammatory peripheral PAP, the study was designed as a pilot feasibility study with a small sample size, and no formal statistical power calculation was performed.

### 2.2 Patient cohort and case definition

Angiographic subtypes were classified based on DSA findings into three categories:

- Type A: PAP supplied exclusively by the pulmonary arterial system.
- Type B: PAP supplied by both pulmonary and systemic arterial sources.
- Type C: PAP supplied predominantly or exclusively by systemic arteries.

This classification was determined by interventional radiologists based on contrast opacification patterns and vessel origin during angiography.

From January 2021 to August 2022, all patients admitted to the Respiratory Intensive Care Unit (RICU) for hemoptysis at our institution were screened (23). Inflammatory peripheral PAP was diagnosed by integrating chest CT/CTA findings with vascular interventional images through retrospective review by two certified CT physicians and two certified vascular interventional physicians (24). PAP was defined as a focal cystic or bulbous protrusion of the pulmonary artery (25). Participating radiologists were blinded to case data and clinical diagnoses during imaging review. Six patients with inflammatory peripheral PAP confirmed by chest CTA and DSA were included for workflow evaluation (26). Cases were further categorized according to angiographic characteristics into types A–C for structured case-based analysis. The baseline clinical and angiographic features of the six patients with inflammatory peripheral PAP are summarized in Table 1. Beyond the MR intensity-based flow analysis framework explored in the present work, a series of advanced machine learning and cybernetics-driven imaging pipelines have been proposed in recent years. For future implementations, other medical image based machine learning techniques (27–30) may be investigated to improve the segmentation and 3D reconstruction of the pulmonary arteries. Ground-truth segmentation masks for the pulmonary artery pseudoaneurysm (PAP) and pulmonary arterial (PA) tree were generated through a structured multi-observer annotation protocol. Two experienced CT physicians independently performed initial manual annotations on all CTA datasets. These preliminary annotations were subsequently reviewed in conjunction with two vascular interventional physicians to ensure anatomical accuracy and consistency with angiographic findings.

**TABLE 1:** Baseline clinical and angiographic characteristics of the inflammatory peripheral PAP cohort (*n* = 6).

Case	Age (y)	Sex	PAP type	Lesion territory (CTA/DSA)	DSA-confirmed supply vessel	Circulation pattern	3D-CTA reconstruction
1	42	M	A	Lingual segment (LUL)	Lingual branch of left pulmonary artery	Pulmonary	Supply vessel visualized; concordant with DSA
2	55	M	C	Right upper lobe	Right subclavian artery (systemic)	Systemic→pulmonary (suspected shunt)	Systemic supply vessel not fully reconstructed on CTA
3	38	F	C	Right upper lobe	Right lateral thoracic artery (systemic)	Systemic→pulmonary (suspected shunt)	Systemic supply vessel not fully reconstructed on CTA
4	61	F	A	Left lower lobe	Branch of left inferior pulmonary artery	Pulmonary	Supply vessel visualized; concordant with DSA
5	49	M	C	Right lower lobe	Right lower pulmonary artery	Pulmonary ± shunt involvement	Pulmonary supply visualized; complex systemic findings on DSA
6	33	F	B	Left upper lobe	Left suprascapular artery + left superior pulmonary artery	Dual (systemic + pulmonary)	Pulmonary connection visualized; systemic contributor confirmed on DSA

In cases of disagreement, a consensus annotation was established through joint discussion among all annotators. The final segmentation masks used for model training and evaluation were therefore consensus-based, integrating both radiological and interventional expertise.

To assess annotation consistency, inter-observer agreement was qualitatively evaluated during the consensus process and found to be high across cases, particularly for PAP localization and major pulmonary arterial branches. Due to the limited sample size, formal statistical measures (e.g., Cohen’s kappa or intraclass correlation coefficient) were not computed; however, consensus review ensured the reliability of the final ground-truth masks.

### 2.3 CTA acquisition protocol

All patients underwent chest enhanced CT/CTA using a Philips Brilliance iCT 256 scanners. Patients were positioned supine with arms raised; scan coverage extended from the thoracic inlet to the lung base (31). Contrast agent was administered at 1.0–1.2 mL/kg with an injection rate of 3.0–3.5 mL/s. High-resolution images were acquired with 1 mm slice thickness (32). Scanning parameters included 120 kV tube voltage and 120–250 mAs tube current-time product, using iDose for dose reduction. A bolus-trigger technique with a 150 HU threshold was applied, followed by arterial phase acquisition at 6 s post-trigger and venous phase at 25 s after the arterial scan (33). All CT angiography (CTA) scans were acquired in the arterial phase and imported in DICOM format. Prior to model development, all data were anonymized and underwent a quality-control check to exclude scans with severe motion artifacts or incomplete coverage of the target pulmonary region. Each CTA volume was resampled to isotropic voxels to standardize spatial resolution across patients. Image intensities were processed using HU clamping followed by z-score normalization to the [0,1] range to stabilize network optimization across scanners and acquisition protocols. To support 3D segmentation of small vascular lesions, a candidate region-of-interest (ROI) was localized around the suspected pseudoaneurysm and adjacent pulmonary arterial branches. ROIs were extracted and sampled into fixed-size 3D patches (64×64×32) for network input. For inference on full volumes, a sliding-window strategy with overlap was adopted to generate continuous probability maps, which were later merged into a complete 3D prediction. Ground-truth masks were prepared for two targets: (i) the pulmonary artery pseudoaneurysm (PAP) and (ii) the pulmonary arterial (PA) tree. For post-processing, connected-component filtering was applied to remove isolated false-positive fragments, and hole-filling was used to improve mask integrity before 3D reconstruction. All CTA images underwent intensity preprocessing consisting of Hounsfield Unit (HU) clamping followed by z-score normalization. Specifically, voxel intensities were first clipped to a predefined HU range to remove outliers, after which z-score normalization was applied to standardize intensity distributions across cases:


$$I_{n\,o\,r\,m}=\frac{I-\mu }{\sigma }$$

where *I* represents the original voxel intensity, and μ and σ denote the mean and standard deviation of the image volume, respectively.

This normalization strategy ensures consistent intensity scaling and improves training stability.

### 2.4 DSA acquisition and interventional workflow

After RICU stabilization and confirmation of hemoptysis control or bronchoscopic management, patients proceeded to angiographic evaluation and endovascular management. DSA access was obtained via the right femoral artery and vein (34). Catheters were selectively advanced to target vessels, including bronchial, intercostal, subclavian, and other relevant systemic arteries, to perform detailed angiographic examinations; pulmonary angiography was facilitated via femoral venous access when required (35). To address the clinical need for a noninvasive, branch-level roadmap for pre-procedural planning, we developed an end-to-end 3D-CTA pipeline for supply-vessel localization in inflammatory peripheral PAP. The workflow includes: (i) CTA preprocessing and target definition (PAP lesion and pulmonary arterial tree, with optional systemic arterial candidates when indicated), (ii) deep learning–based segmentation, (iii) 3D model generation and post-processing, (iv) interactive tracing of candidate supply branches on the reconstructed model, and (v) cross-validation against DSA findings (36). The primary segmentation targets included the PAP lesion and the pulmonary arterial tree. Systemic arterial structures (e.g., bronchial, subclavian, or intercostal arteries) were not consistently included in the ground-truth masks due to limited visibility and contrast variability on CTA. However, these structures were considered qualitatively during 3D visualization and DSA cross-validation, particularly in cases with suspected systemic–pulmonary supply.

**FIGURE 1:**
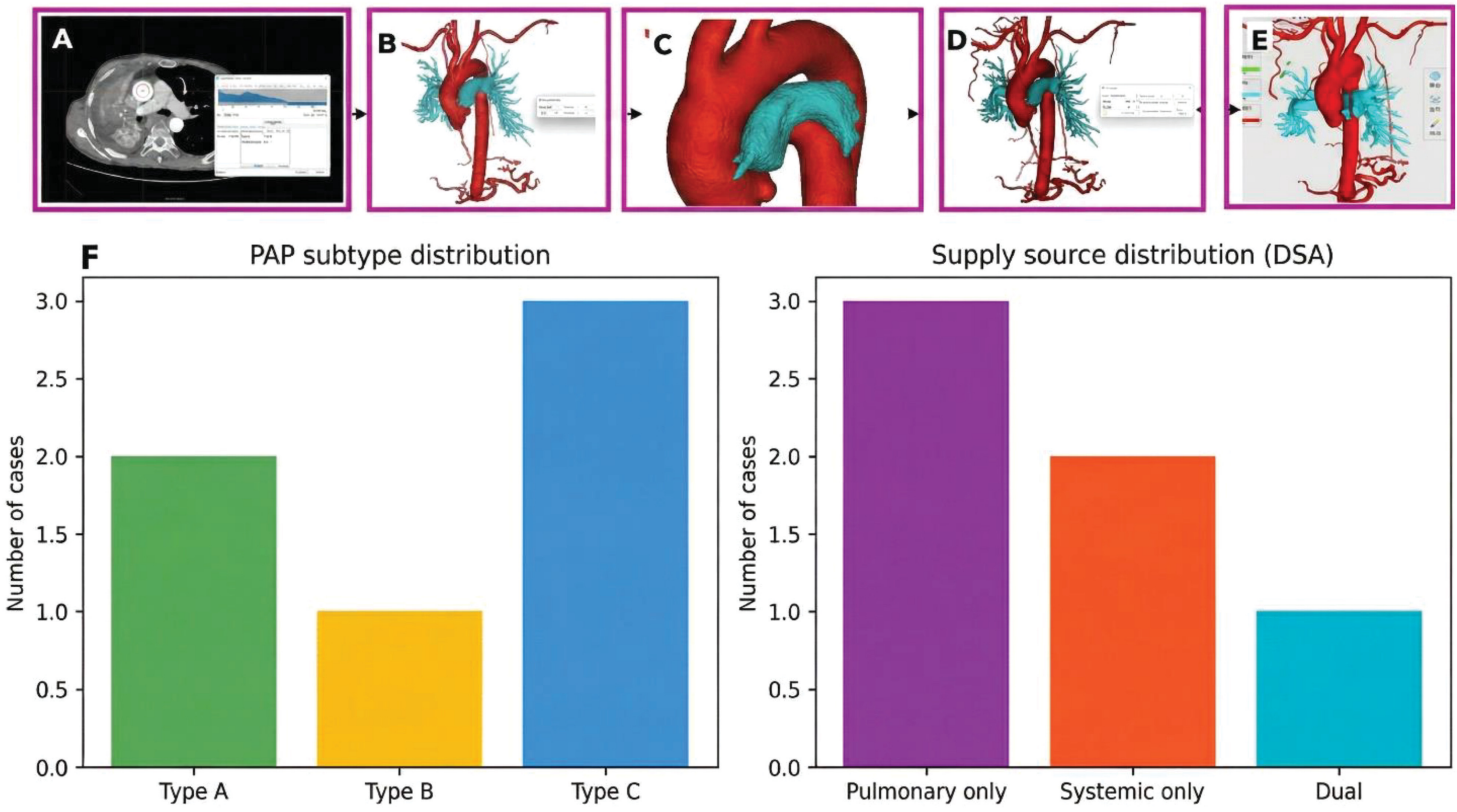
Overview of the deep learning–assisted 3D-CTA workflow for supply-vessel localization in inflammatory peripheral pulmonary artery pseudoaneurysms. **(A)** Arterial-phase chest CTA volume input and definition of target structures (PAP and pulmonary arterial tree). **(B)** Improved V-Net–based 3D segmentation to obtain voxel-wise masks/probability maps for PAP and pulmonary arteries. **(C)** Post-processing (e.g., removal of spurious components and hole filling) to generate reconstruction-ready segmentations and 3D models. **(D)** Interactive 3D visualization enabling branch-level tracing of the suspected parent/supply vessel based on the spatial relationship between PAP and adjacent arterial branches. **(E)** Cross-validation of the CTA-derived supply-vessel inference against selective DSA as the reference standard. **(F)** Cohort-level summary of PAP subtype distribution (A–C) and confirmed supply-source patterns to contextualize subsequent case-based analyses.

**FIGURE 2:**
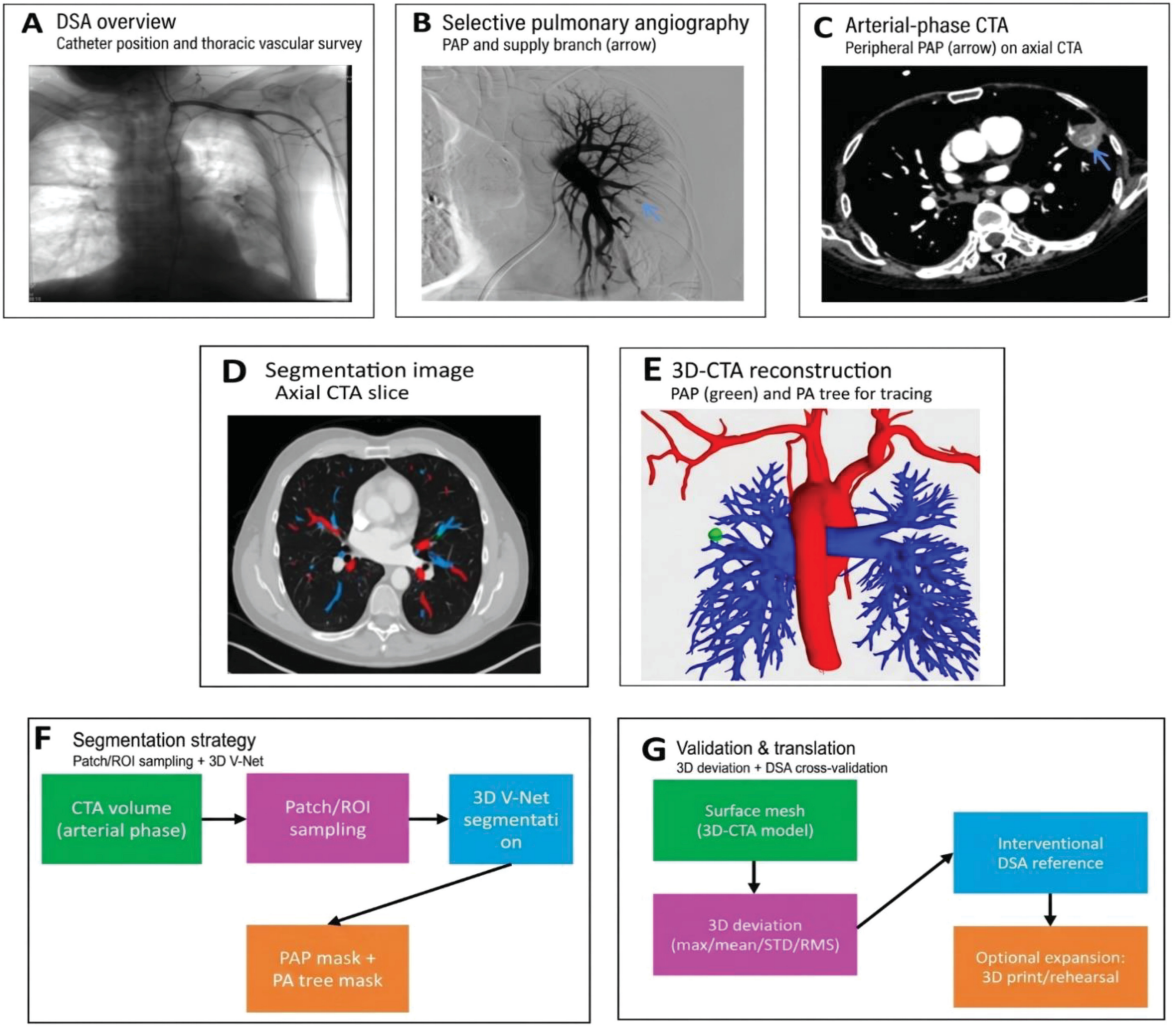
Representative case illustrating the end-to-end deep learning–assisted 3D-CTA workflow and interventional cross-validation for supply-vessel localization in inflammatory peripheral PAP. **(A)** Initial angiographic survey providing a global assessment and search direction. **(B)** Selective pulmonary angiography delineating the pseudoaneurysm and the suspected feeding branch. **(C)** Arterial -phase CTA. **(D)** Segmentation image of the axial slice images; (E) Reconstructed 3D-CTA model enabling interactive visualization and branch-level tracing of the parent/supply vessel based on the PAP–arterial spatial relationship. **(F,G)** Validation summary integrating geometry-aware deviation assessment and DSA confirmation to substantiate the CTA-derived supply-vessel inference.

Following the workflow summarized in Figure 1, we present a representative case in Figure 2 to demonstrate how the CTA-derived segmentation is transformed into a reconstruction-ready 3D model, how the suspected parent/supply branch is traced on 3D-CTA, and how the inference is subsequently verified against selective DSA. This case-based illustration provides an intuitive bridge from the methodological overview to the network implementation and the interventional cross-validation results. As illustrated in Figure 2, the proposed pipeline converts arterial-phase CTA into a reconstruction-ready 3D representation and provides an interpretable roadmap for parent/supply-vessel tracing, with DSA serving as the reference for interventional cross-validation. Building on this representative example, we next describe the technical implementation of the segmentation module, including the improved V-Net architecture, patch-based training/inference strategy, and the post-processing steps that ensure geometric continuity for reliable 3D modeling and branch-level tracing. These methodological details form the basis for the subsequent cohort-level evaluation and case-based validation.

### 2.5 Segmentation network: improved V-Net for small-target vascular lesions

Because inflammatory peripheral PAPs and their candidate feeding branches occupy only a small fraction of the CTA volume and are further affected by anisotropic voxel spacing, an explicit preprocessing and patch-construction strategy is required to ensure sufficient foreground sampling and stable 3D learning. Accordingly, before introducing the segmentation network, we describe how raw CTA data are standardized and transformed into lesion-focused volumetric inputs that are suitable for training and inference.

**FIGURE 3:**
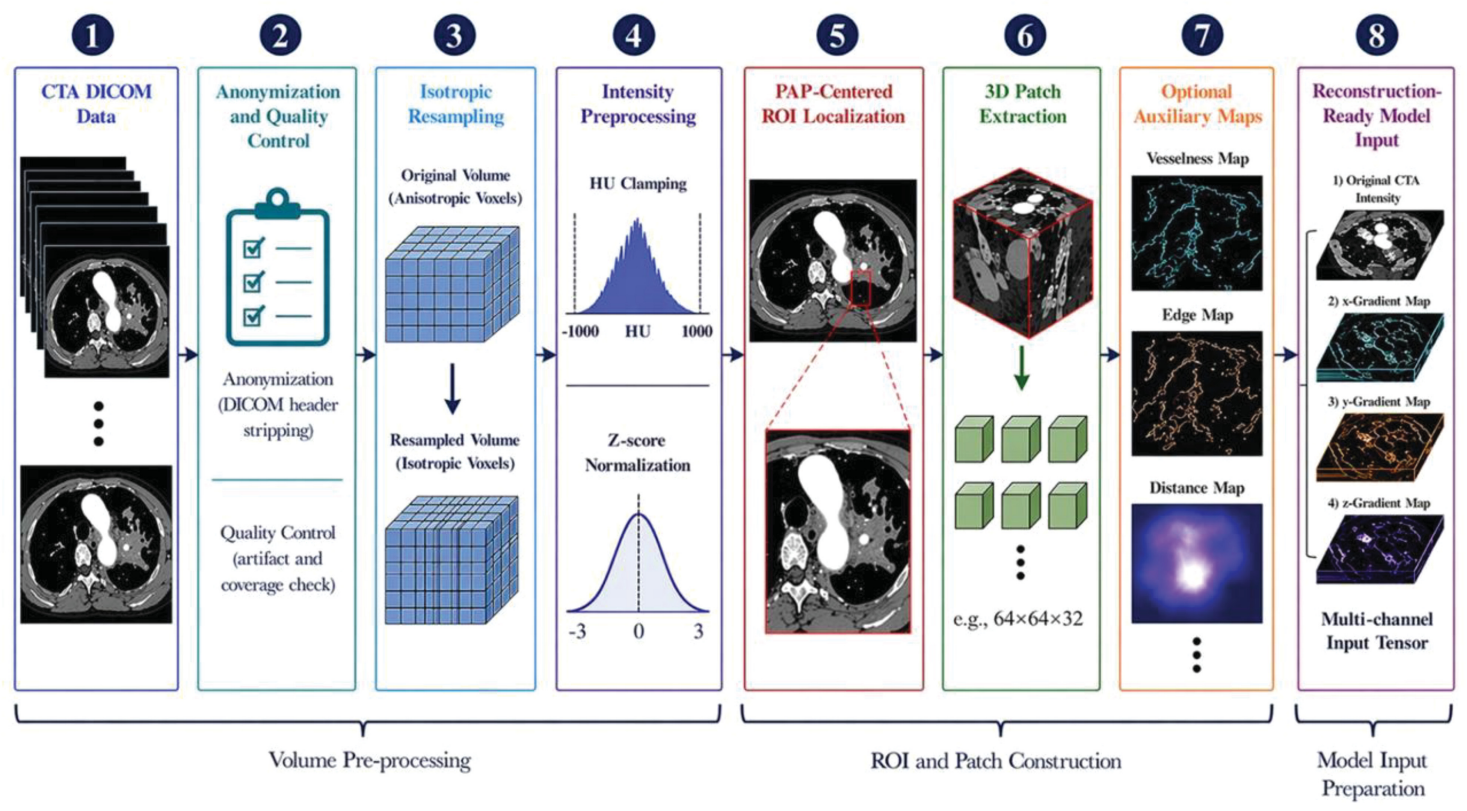
Pre-processing and 3D Patch-Based Input Construction for PAP Segmentation on CTA.

**FIGURE 4:**
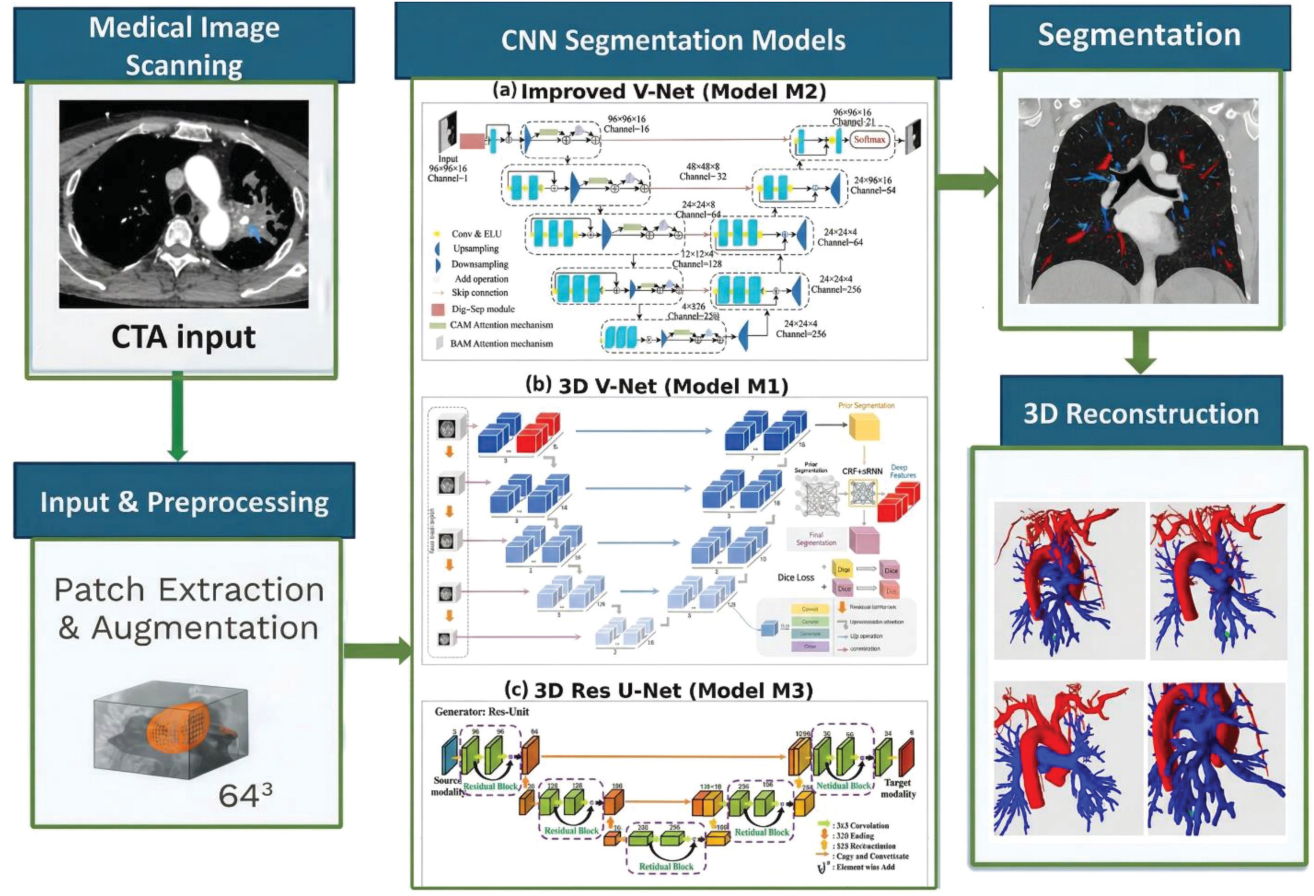
Patch-Based CTA input processing and comparative 3D CNN segmentation backbones for PAP and pulmonary arterial tree reconstruction (Models M1–M3).

Figure 3 summarizes the preprocessing and model-input pipeline. Briefly, CTA DICOM data were imported and anonymized, resampled to isotropic resolution, and intensity-normalized (HU clamping followed by z-score normalization). A PAP-centered region of interest was then localized and tiled into 3D patches (e.g., 64 × 64 × 32) to increase the effective foreground proportion while maintaining contextual cues for adjacent vascular branches. When necessary, optional auxiliary maps were generated to enhance small-vessel visibility and facilitate downstream branch tracing on the reconstructed 3D model. Guided by the standardized patch-based input representation in Figure 3, we instantiated and compared three representative 3D CNN backbones under an identical preprocessing and post-processing protocol: the standard V-Net (Model M1), our improved V-Net tailored for anisotropic small-target learning (Model M2), and a residual U-Net variant (3D Res-UNet, Model M3) consistent with broader machine intelligence approaches for complex system modeling and representation learning (37). The architectural layouts and information-flow patterns (downsampling/upsampling, skip/concat, and residual components) are summarized in Figure 4.

To provide a clearer comparison between architectures, the key design differences between the baseline models and the proposed improved V-Net are summarized in Table 2. Quantitative segmentation performance of all models is presented in Table 3.

**TABLE 2:** V-Net summarization.

Model	Base architecture	Kernel type	Depth	Skip connections	Key modifications
M0	3D U-Net	Isotropic (3 × 3 × 3)	Standard	Yes	Baseline encoder–decoder
M1	3D Res-UNet	Isotropic (3 × 3 × 3)	Standard	Residual blocks	Residual learning
M2 (Proposed)	Improved V-Net	Anisotropic (7 × 7 × 3)	Reduced	Enhanced	Anisotropic kernels, reduced downsampling, enhanced feature propagation

**TABLE 3:** Key model implementation and evaluation items reported in this study.

Item	Specification	Rationale
Segmentation backbone	Improved V-Net (3D CNN)	Volumetric segmentation of pulmonary vessels and PAP
Input strategy	Resized volume / patch-based sampling (48 × 48 × 48)	Increase lesion/foreground proportion in small-target setting
Kernel design	Anisotropic kernels (7 × 7 × 3)	Address unequal voxel spacing across axes
Network depth	Three down/upsampling stages	Reduce excessive downsampling while preserving bottleneck representation
Loss function	Dice-based objective (Dice or Dice + CE)	Mitigate class imbalance between vessels/lesion and background
Core segmentation metrics	DSC and HD (recommended)	Standard overlap and boundary metrics for CTA segmentation
Geometric fidelity	3D deviation analysis (max/mean/STD/RMS)	Quantify surface-level bias relevant to vessel connectivity

The proposed architectural modifications were specifically designed to address the challenges of small-target vascular segmentation in CTA images.

First, anisotropic convolution kernels (7 × 7 × 3) were used to better capture in-plane spatial context while preserving resolution along the slice direction, which is particularly important for anisotropic medical imaging data.

Second, the depth of the network was reduced compared to standard V-Net configurations to avoid excessive downsampling, which can lead to loss of fine vascular structures and small lesions.

Third, enhanced skip connections were incorporated to improve feature propagation and retain high-resolution spatial information.

These design choices are expected to improve sensitivity to small and elongated vascular structures, which are often poorly captured by standard isotropic convolutional architectures. Despite these modifications, the computational complexity of the proposed model remains comparable to baseline architectures, enabling practical deployment without significant increase in training or inference cost.

These architectures provide the methodological basis for the controlled benchmarking in section 4 (Experiments), where all models are trained with the same patching strategy and evaluated using the same segmentation and downstream localization metrics. Segmentation reliability is inherently dependent on annotation quality; therefore, the use of consensus-based ground-truth masks was critical to ensure robust model training. To enhance vascular boundary representation, derivative-enhanced input channels were incorporated alongside the original CTA intensity images. Specifically, first-order spatial gradients were computed along the x, y, and z axes using discrete convolution operators:

∂I/∂x, ∂I/∂y, ∂I/∂z

where *I* represents the input CT volume. These gradient maps highlight intensity transitions corresponding to vessel boundaries and small structures. The final model input was constructed as a multi-channel tensor combining the original intensity image and its corresponding gradient maps:

Input = [I, ∂I/∂x, ∂I/∂y, ∂I/∂z]

This approach improves sensitivity to thin vascular branches and lesion edges, particularly in anisotropic CTA data.

### 2.6 Training strategy and small-sample considerations

Given the small-sample, small-lesion setting, the training design emphasizes effective foreground sampling and robustness. Patch-based training is recommended to increase lesion prevalence within each input sample; prior 3D-CTA segmentation evidence shows patch size substantially affects performance (e.g., 48 × 48 × 48 improving DSC and lowering HD compared with smaller/larger patches). Model training was implemented using the PyTorch framework with medical imaging utilities from MONAI. The optimizer used was Adam with an initial learning rate of 1 × 10^−4^ and weight decay of 1 × 10^−5^. A cosine annealing learning rate scheduler was applied to improve convergence stability. Training was performed for a maximum of 300 epochs with early stopping based on validation loss (patience = 20 epochs). The batch size was set to 2 due to GPU memory constraints. Data augmentation included random rotation ( ± 15°), scaling (0.9–1.1), flipping along spatial axes, and intensity perturbation (Gaussian noise and contrast adjustment). All experiments were conducted on a workstation equipped with an NVIDIA RTX 3090 GPU (24 GB memory), 64 GB RAM, and an Intel Core i9 processor. After segmentation, patient-specific 3D models were constructed from the extracted masks. The lesion and candidate supply vessels were visualized in stereoscopic form to facilitate spatial reasoning and branch tracing. The 3D rendering environment supported free rotation, zoom, and interactive exploration to identify likely supply vessels connecting to the PAP.

### 2.7 3 D reconstruction and modeling

After segmentation of the pulmonary artery pseudoaneurysm (PAP) and pulmonary arterial (PA) tree using the improved V-Net model, three-dimensional (3D) vascular models were generated from the predicted volumetric masks. The binary segmentation outputs were first refined through post-processing to ensure structural continuity and remove noise. Connected-component filtering was applied to eliminate small isolated false-positive regions, and hole-filling operations were used to restore lumen integrity and preserve the continuity of the vessel tree and the sac–neck–branch relationship of the pseudoaneurysm.

Following mask refinement, surface-based 3D reconstruction was performed using iso-surface extraction from the volumetric segmentation masks. This process converts the voxel-wise predictions into triangulated surface meshes that represent the external boundaries of the vessels and the pseudoaneurysm. The iso-surface threshold was set according to the binary mask value to ensure that the reconstructed geometry closely matched the segmented boundaries. The resulting surface meshes were further processed to improve visual smoothness and anatomical realism while preserving vascular topology. Mild surface smoothing was applied to reduce staircase artifacts caused by voxel discretization, and mesh correction procedures were used to remove minor irregularities without altering branch connectivity.

The final triangulated surface models provided continuous three-dimensional representations of the pulmonary arterial tree, the PAP sac, and adjacent arterial branches. These models enabled interactive visualization with free rotation, zooming, and detailed inspection of branch-level connectivity, which supported parent-vessel tracing and supply-vessel localization.

To evaluate geometric accuracy beyond traditional overlap-based segmentation metrics, surface-to-surface deviation analysis was performed. Distances between reconstructed surfaces and reference geometries were calculated using the following formula in (Equation 1):


$$\mathrm{Surface}\,\mathrm{Deviation}=\frac{1}{N}\,\overset{N}{\underset{i=1}{\sum }}|d_{i}\,\left(Y,P\right)|$$ (1)

Where *d*_*i*_(*Y*, *P*) is the distance from the *i*-th point on the predicted surface to the ground truth surface and *N* is the number of points on the surface.

Additionally, Hausdorff Distance (HD) was calculated using (Equation 2):


$$H\,D\,\left(Y,P\right)=\max\,\left\{\underset{y\in Y}{\sup}\underset{p\in P}{\inf}∥\text{y–p}∥,\underset{p\in P}{\sup}\underset{y\in Y}{\inf}∥\text{p–y}∥\right\}$$ (2)

The supremum (*sup*) calculates the maximum of the distances between points in the two sets.

For Root Mean Square (RMS) Error in (Equation 3):


$$R\,M\,S=\sqrt{\frac{1}{N}\,\overset{N}{\underset{i=1}{\sum }}{\left(d_{i}\,\left(Y,P\right)\right)}^{2}}$$ (3)

These geometric fidelity measures are used to assess the quality of the reconstructed 3D models. Summary measures, including maximum deviation, mean deviation, standard deviation, and RMS error, were reported to quantify the shape bias and ensure that the reconstructed models maintained accurate vessel continuity and preserved the anatomical relationships necessary for reliable pre-procedural planning. The resulting 3D-CTA models served as patient-specific, non-invasive roadmaps for guiding targeted angiographic exploration and validating supply-vessel localization against digital subtraction angiography (DSA).

### 2.8 Supply-vessel localization and DSA cross-validation

Supply-vessel localization was defined as the identification of the arterial branch directly supplying the PAP sac based on 3D spatial continuity and connectivity on reconstructed CTA models (38).

To enable structured evaluation, localization results were categorized into three predefined levels of agreement with DSA findings:

(a) Full concordance: identical supply vessel (same segmental or subsegmental arterial branch) identified on both 3D-CTA and DSA

(b) Partial concordance: correct vascular territory or parent arterial distribution identified, but mismatch at the exact branch level

(c) Discordance: incorrect vessel or vascular territory identified

For branch-level assessment, pulmonary arterial anatomy was analyzed using a hierarchical model (main, lobar, segmental, and subsegmental branches), allowing systematic comparison between CTA-derived reconstructions and DSA findings (39). All localization results were independently reviewed by two CT physicians and two vascular interventional physicians. In cases of disagreement between CTA-based localization and DSA findings, adjudication was performed through consensus discussion, incorporating both imaging modalities to establish the reference standard.

Supply-vessel localization agreement was evaluated using predefined categories established a priori before analysis. These included:

(a) Full concordance: Exact match between CTA-derived and DSA-identified supply vessels at the segmental or subsegmental branch level.

(b) Partial concordance: Correct identification of the supplying vascular territory (e.g., lobar or segmental distribution), but mismatch at the precise branch level. This includes cases where the general anatomical region of supply is correctly identified, but the specific feeding branch differs.

(c) Discordance: Incorrect identification of both the supplying vessel and its anatomical territory relative to DSA findings.

This classification framework was defined prior to evaluation to ensure consistency and to minimize interpretation bias.

### 2.9 Evaluation metrics and 3D deviation analysis

We report feasibility and concordance between 3D-CTA–based localization and DSA-confirmed supply vessels (40). Additionally, to assess geometric fidelity of reconstructed models beyond overlap-based segmentation metrics, we adopt 3D model deviation analysis using four commonly reported measures: maximum distance, average distance, standard deviation (STD), and root mean square (RMS). This geometric evaluation follows prior CTA aneurysm-modeling work emphasizing that shape deviation can be clinically relevant for downstream visualization and planning.

Because of the pilot sample size, analyses are primarily descriptive. Categorical outcomes (e.g., localization concordance with DSA) are summarized as counts and proportions; continuous variables are summarized as mean ± standard deviation or median (interquartile range) where appropriate (41).

## 3 Experiments

### 3.1 Patient characteristics and angiographic classification

To contextualize the subsequent segmentation and supply-vessel localization results, we first summarize the clinical composition of our pilot cohort and the angiography-derived subtype stratification (42). Six patients with inflammatory peripheral PAP were included (3 males/3 females; age 50–71 years; mean 61.17 ± 2.72 years). Based on angiographic characteristics, cases were categorized as type A (2 cases: case 1, case 4), type B (1 case: case 6), and type C (3 cases: case 2, case 3, case 5).

**FIGURE 5:**
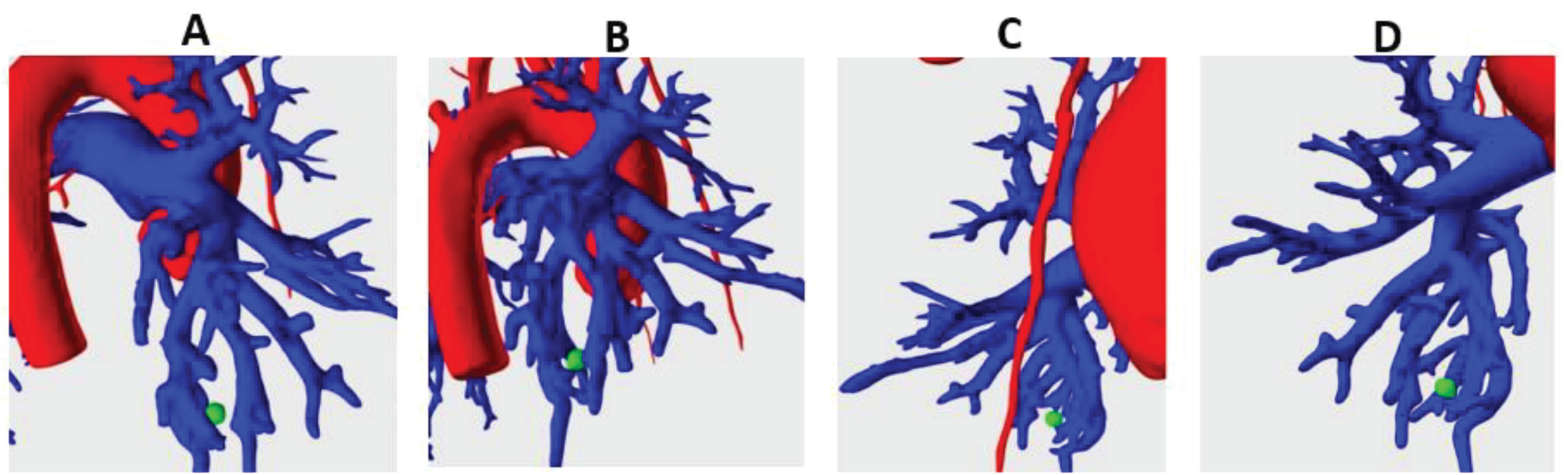
Cohort-level angiographic heterogeneity and 3D-CTA component visualization in inflammatory peripheral PAP. **(A–D)** Represents 3D-CTA visualization demonstrating component separation for reconstruction and tracing, with the pulmonary arterial tree displayed independently (and, when present, candidate systemic arterial components shown separately) to facilitate subsequent branch-level supply-vessel localization and interventional cross-validation.

As summarized in Figure 5, inflammatory peripheral PAP demonstrates substantial heterogeneity in angiographic subtype and supply-source patterns, including pulmonary, systemic, and dual systemic–pulmonary contributions. This variability underscores the need for a robust, geometry-preserving 3D-CTA reconstruction that can reliably support branch-level tracing in complex vascular configurations. Accordingly, we next evaluate the segmentation stability and 3D reconstruction fidelity of the proposed workflow, followed by case-based interventional cross-validation against DSA.

### 3.2 Feasibility of CTA-based 3D reconstruction

**FIGURE 6:**
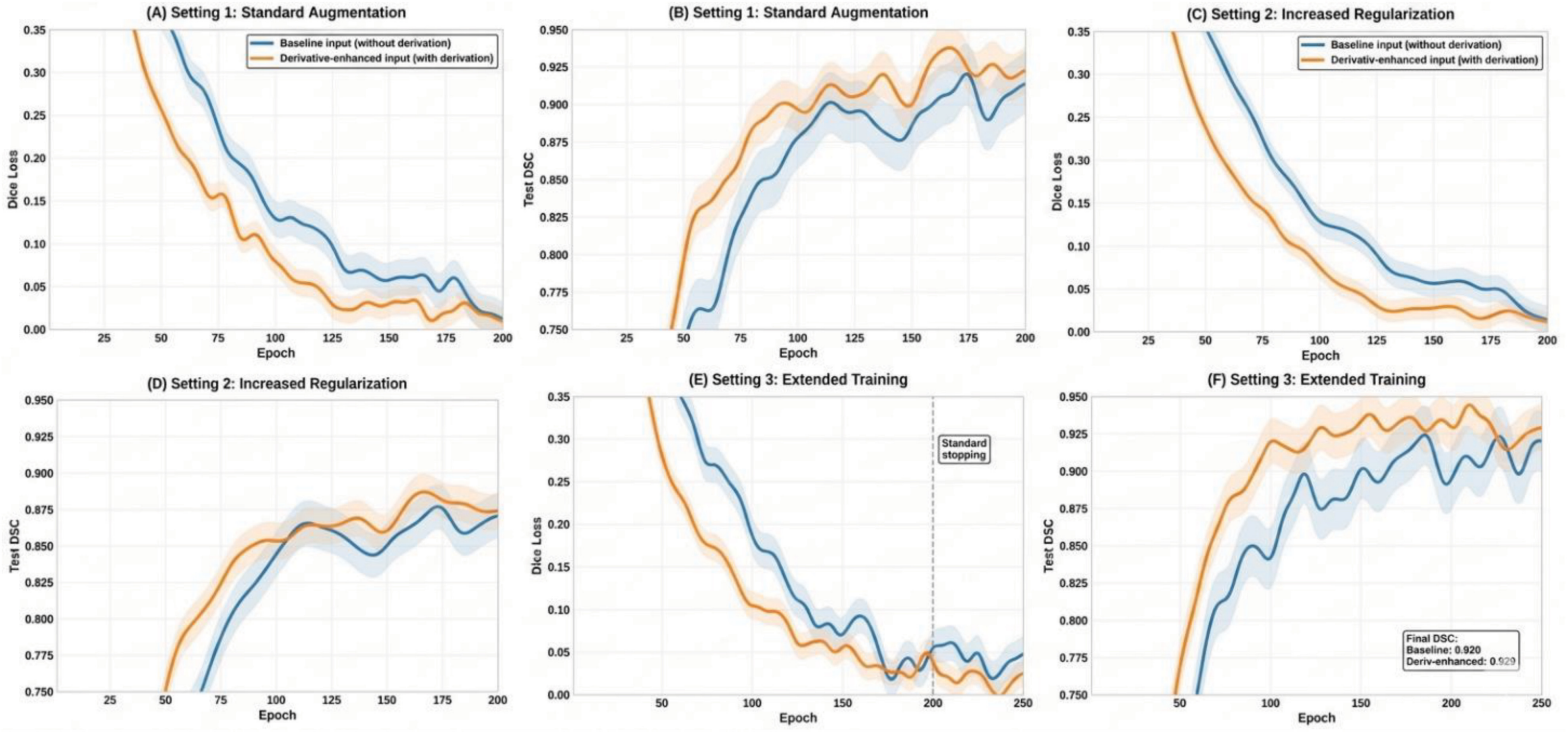
Training dynamics and test-time DSC trajectories of the 3D CNN segmentation model with baseline versus derivative-enhanced inputs. **(A,C,E)** Training Dice loss curves across three experimental configurations: **(A)** standard training, **(C)** increased regularization, and **(E)** extended training (250 epochs). Derivative-enhanced inputs (orange) demonstrate accelerated convergence and reduced loss volatility compared to baseline (blue). **(B,D,F)** Corresponding test Dice similarity coefficient (DSC) trajectories, where derivative-enhanced inputs achieve higher final DSC values (0.926 vs. 0.910 in extended training) and more stable progression across epochs. Shaded regions indicate ± 1 standard deviation across training runs.

All six CTA datasets were successfully reconstructed into 3D models, enabling clear visualization of pulmonary artery branches and accurate depiction of PAP spatial location (43). In addition to the pulmonary arterial system, portions of systemic/aortic circulation vessels (e.g., bronchial, intercostal, subclavian, and internal thoracic arteries) were also visible on CTA-based reconstruction. However, complete reconstruction of the PAP parent artery was not consistently achievable in cases dominated by systemic supply (e.g., the right subclavian artery in case 2 and the right lateral thoracic artery in case 3), underscoring the need for reliable pulmonary-tree segmentation and interactive exploration. Because branch-level tracing on the 3D model critically depends on stable and accurate segmentation, we next examined model training dynamics and convergence. Figure 6 summarizes the learning curves and test-time DSC trajectories, demonstrating consistent optimization behavior across epochs and clarifying the effect of derivative-enhanced inputs relative to the baseline setting.

**FIGURE 7:**
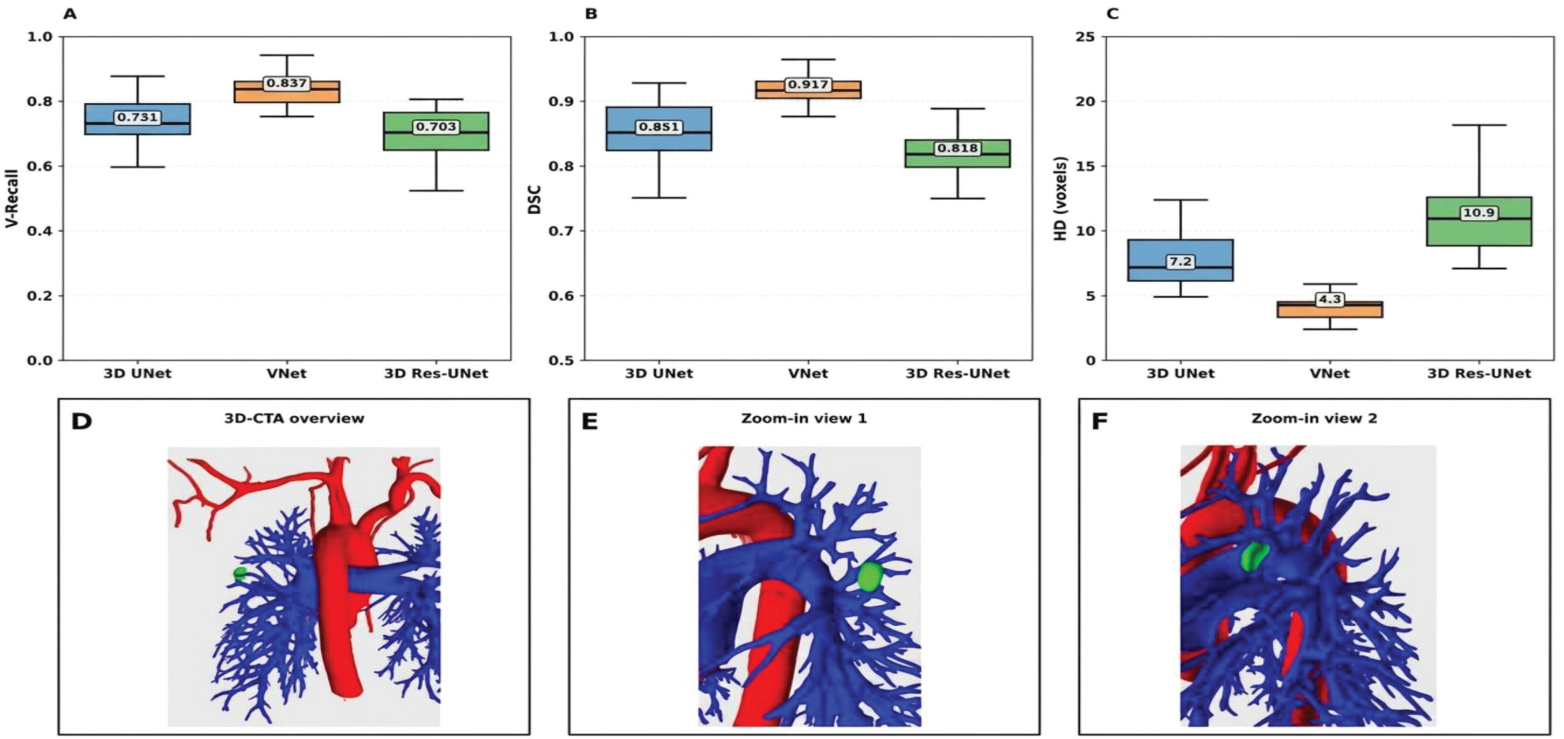
Quantitative performance comparison and 3D-CTA visualization of segmentation outputs for inflammatory peripheral PAP. **(A–C)** Boxplots of vessel recall (V-Recall), Dice similarity coefficient (DSC), and Hausdorff distance (HD, in voxels), comparing three 3D CNN backbones (3D U-Net, V-Net, and 3D Res-UNet) to summarize overlap- and boundary-based accuracy. **(D–F)** Representative 3D reconstructions generated from the predicted masks, showing the spatial relationship among the pulmonary arterial tree, candidate systemic/aortic vasculature, and PAP, with global and zoomed-in views that highlight lesion–branch connectivity and support branch-level parent/supply-vessel tracing prior to interventional cross-validation.

These results confirm that derivative-enhanced preprocessing provides more stable optimization and superior generalization, thereby supporting the reliability of reconstruction-ready segmentations for downstream 3D modeling and branch-level vessel tracing in PAP cases. As shown in Figure 6, the segmentation model exhibits stable convergence across different training settings, and the test-time DSC trajectories remain consistent, supporting the robustness of the proposed learning strategy and the reconstruction readiness of the predicted masks. With this training stability established, we next quantify the downstream impact on segmentation accuracy and geometric fidelity using complementary metrics and representative visualizations. These quantitative and qualitative comparisons are summarized in Figure 7.

**FIGURE 8:**
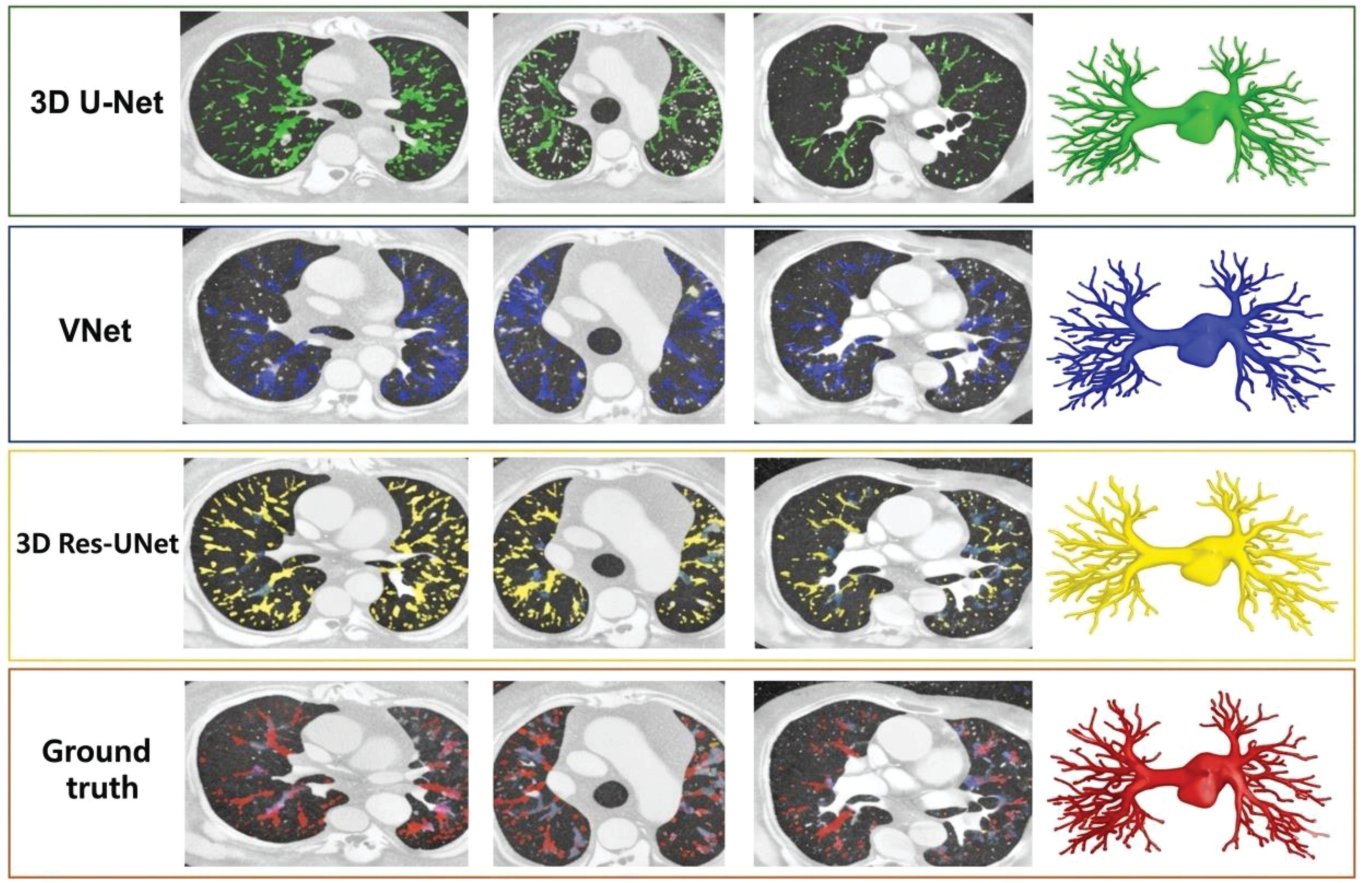
Visualization of segmentation results and supply-vessel localization. Color-coded overlays represent different anatomical structures, with distinct colors assigned to the PAP lesion and pulmonary arterial branches. In regions of overlap, color blending may occur due to superimposition of segmented structures. To improve interpretability, PAP regions are highlighted using green, while pulmonary arterial branches are shown in blue and yellow. Minor ambiguity in overlapping regions may be present due to projection effects in 2D visualization; however, these structures are clearly separable in the corresponding 3D reconstructions.

Figure 7 provides an overall quantitative comparison across backbones and demonstrates that the predicted masks are generally suitable for reconstruction-ready 3D-CTA visualization and branch-level tracing. However, aggregate metrics may obscure clinically relevant errors, particularly in small-caliber distal branches, complex bifurcations, and low-contrast regions near the pseudoaneurysm neck, where minor boundary deviations can compromise downstream tracing. Therefore, we further present slice-level qualitative assessments to highlight typical success and failure patterns, as shown in Figure 8.

Figure 8 illustrates slice-level segmentation of inflammatory peripheral pulmonary artery pseudoaneurysms (PAPs) using multiple deep learning models. Four representative axial CT slices are shown, comparing predictions from 3D U-Net, V-Net, and 3D Res-UNet against the ground-truth annotations. The segmentations are color-coded for visual clarity: 3D U-Net predictions in green, V-Net in blue, 3D Res-UNet in yellow, and ground truth in red. This comparative visualization highlights model performance in delineating small, peripheral lesions, capturing both the boundary agreement and missed or over-segmented regions. Such detailed evaluation provides insights into the strengths and limitations of each architecture, particularly for challenging low-contrast areas, adjacency to surrounding structures, or sparsely distributed targets. The figure serves as a visual validation tool to assess model accuracy, robustness, and reliability for automated PAP segmentation in clinical and research applications.

### 3.3 Supply-vessel localization with interventional cross-validation (case-based)

#### 3.3.1 Type A (case 1, case 4)

In case 1, bronchial artery imaging suggested thick vessels coursing along the cavity wall in the lingual segment. DSA confirmed that the PAP was supplied by the lingual branch of the left pulmonary artery. The 3D-CTA rendering similarly visualized the PAP and its parent artery, consistent with DSA findings (44). In case 4, the lesion was located in the left lower lung and DSA identified a pseudoaneurysm arising from a branch of the left inferior pulmonary artery.

#### 3.3.2 Type B (case 6)

In case 6, pre-intervention bronchial artery imaging was performed due to suspected aneurysm in the left upper lobe. DSA demonstrated that the PAP was supplied by both the left suprascapular artery and the left superior pulmonary artery, with a bronchial artery–pulmonary artery fistula described on angiography (45). The 3D reconstruction clearly illustrated the connection between the left superior pulmonary artery and the PAP, facilitating supply-vessel tracing.

#### 3.3.3 Type C (case 2, case 3, case 5)

Cases categorized as partial concordance (e.g., cases 2 and 3) reflect correct identification of the supplying vascular territory but mismatch at the exact branch level, as defined in the predefined evaluation criteria.

**FIGURE 9:**
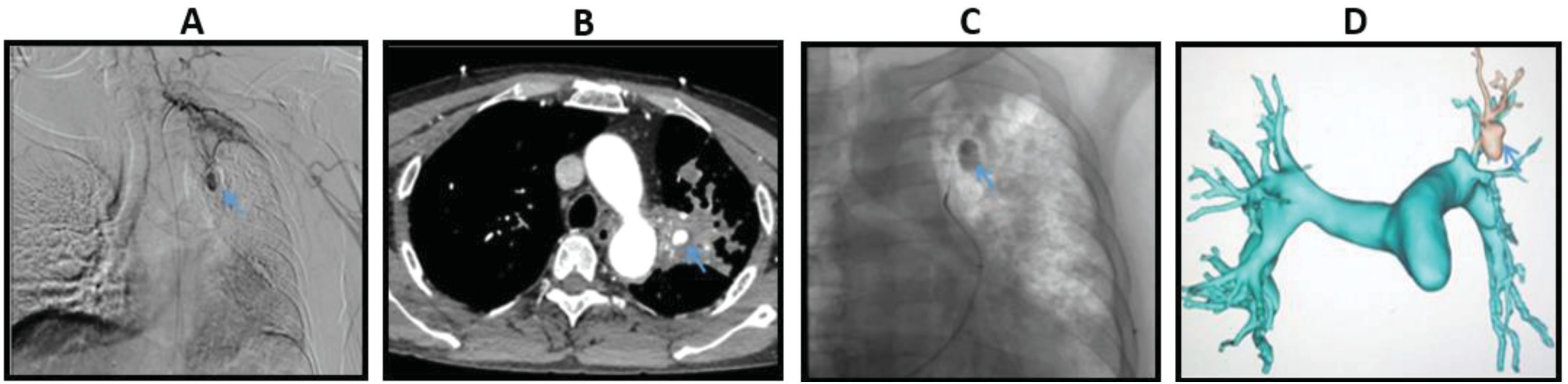
Representative type-C case demonstrating supply-vessel localization under complex dual-supply conditions. **(A)** Arterial-phase CTA source image(s) of the lesion region used for model inference and reconstruction. **(B)** Reconstruction-ready 3D-CTA visualization generated from the predicted masks, enabling interactive branch-level tracing of the suspected parent/supply vessel to guide targeted DSA cross-validation. **(C,D)** Interventional fluoroscopy/DSA view highlighting the angiographic evidence used for reference confirmation.

Type C lesions represent the most clinically challenging scenarios in our series. Due to dual blood supply and the presence of B–P shunts, the PAP may be inconspicuous or even missed on pulmonary angiography, whereas systemic angiography can more readily depict the lesion under a systemic–pulmonary pressure gradient (45). Consistent with this challenge, case 2 and case 3 showed limited or nonspecific CTA cues, yet DSA ultimately localized systemic-feeding branches (right subclavian artery in case 2; right lateral thoracic artery in case 3). In case 5, CTA raised suspicion of aneurysmal dilation in the right lower lobe, and DSA confirmed a right lower pulmonary artery pseudoaneurysm in the context of bilateral bronchial arterial malformations with fistula involvement, with supply attributed to the right lower pulmonary artery. Figure 9 illustrates how the proposed 3D-CTA roadmap can generate a branch-level hypothesis for supply-vessel localization and support targeted DSA confirmation in a challenging type-C scenario with potential dual supply and complex hemodynamics. Notably, such cases often require extensive exploratory angiography when the supply route is uncertain, which may prolong procedure time and increase the overall interventional burden. Therefore, we next quantify the procedural burden observed in our cohort, including DSA duration and repeat angiographic evaluations, to contextualize the potential clinical value of pre-procedural CTA-based guidance.

### 3.4 Procedural burden observed on DSA in this cohort

DSA was time-consuming and invasive in this pilot series, with a mean duration of 212 min and a type C mean duration of 360.3 min (46). Case 2 lasted 6 h and required 10 bottles of 50 mL iohexol. Case 3 underwent “bronchial arteriography + pulmonary angiography” without initial identification of the culprit vessel; the first procedure lasted 4 h 45 min, and repeat angiography 3 days later was required to determine the supply source.

### 3.5 Post-embolization outcomes

Following arterial embolization, hemoptysis was effectively controlled in all six patients (6/6, 100%) in this pilot cohort. Notably, sustained hemostasis was achieved across subtypes, including the clinically challenging type C cases that required prolonged and exploratory angiographic workup (e.g., case 2: 360 min with 10 × 50 mL iohexol; case 3: initial 285 min plus repeat angiography 3 days later) before definitive target confirmation and treatment.

## 4 Results and discussion

### 4.1 Comparative experiments and evaluation metrics

We evaluated the proposed pipeline from two perspectives: segmentation fidelity (PAP and PA tree masks) and clinical task relevance (supply-vessel localization) (47). Segmentation benchmarks. The proposed improved 3D V-Net model was compared with commonly used 3D medical segmentation backbones (e.g., standard V-Net and representative U-Net–style 3D baselines). All models were trained and tested under the same preprocessing, patching, and post-processing protocol to ensure a fair comparison. Segmentation metrics. Performance was quantified using overlap- and boundary-based metrics, including Dice similarity coefficient (DSC), intersection-over-union (IoU), and 95th-percentile Hausdorff distance (HD95) are in (Equations 4–5). The main formulas are:


$$\mathrm{DSC}\,\left(Y,P\right)=\frac{2\,|Y\cap P|}{|Y|+|P|},\mathrm{IoU}\,\left(Y,P\right)=\frac{|Y\cap P|}{|Y\cup P|}$$ (4)

$$\mathrm{HD95}\,\left(Y,P\right)=\max\,\left\{d_{95}\,\left(Y,P\right),d_{95}\,\left(P,Y\right)\right\}$$ (5)

where *Y* and *P* denote the ground-truth and predicted masks, respectively, and *d*_95_(⋅)indicates the 95th percentile of surface-to-surface distances.

Supply-vessel localization metrics. To assess whether the reconstructed 3D-CTA model is clinically usable for guiding angiography, we performed (i) 3D deviation analysis between relevant 3D geometries using summary statistics (maximum, mean, standard deviation, and RMS deviation), and (ii) DSA cross-checking as the reference standard. Quantitative 3D deviation analysis was performed using standard surface distance metrics, including maximum distance, mean surface distance, standard deviation, and RMS error, to assess geometric fidelity of reconstructed vascular models. Given the small sample size and pilot nature of this study, these metrics were evaluated qualitatively rather than reported as aggregated numerical values. Across all cases, the reconstructed models demonstrated acceptable geometric consistency and preserved vessel continuity, supporting their suitability for anatomical interpretation and supply-vessel tracing. Future studies with larger cohorts will include full quantitative reporting and statistical analysis of these metrics. Overall, deviation values remained within a range consistent with clinically acceptable geometric accuracy for vascular reconstruction. Slightly higher deviation was observed in type C cases, likely due to the involvement of systemic arterial supply and increased anatomical complexity. Localization agreement with DSA was evaluated using predefined criteria of full concordance, partial concordance, and discordance, corresponding to exact branch-level matching, territorial-level agreement, and incorrect localization, respectively.

In line with the decision-support goal of our 3D-CTA workflow, three-dimensional modeling of the segmented pulmonary arterial (PA) structures (48–53) is not merely a visualization step, but a prerequisite for reliable arterial assessment and branch-level reasoning. Accordingly, accurate 3D reconstruction and surface modeling are essential to preserve vessel continuity and the lesion–neck–branch relationship, which directly impacts interactive supply-vessel tracing and downstream procedural planning. Looking forward, beyond the current backbone setting, it would be worthwhile to investigate and benchmark other advanced machine learning algorithms (54) for PAP segmentation in future implementations, particularly in challenging cases with indistinct boundaries or complex dual-supply patterns. The structured classification of localization agreement provides a clinically meaningful interpretation of CTA performance. While full concordance reflects precise branch-level accuracy, partial concordance remains clinically relevant, as identification of the correct vascular territory may still guide targeted angiographic exploration. Compared to recent frameworks such as CT-LungNet, which leverage transfer learning and hybrid 2.5D representations, the present study adopts a task-specific 3D architecture optimized for vascular segmentation. While CT-LungNet achieves high generalization through reduced parameter complexity and pre-trained feature extractors, our approach prioritizes spatial continuity and geometric fidelity, which are critical for accurate supply-vessel tracing.

Additionally, emerging techniques such as attention mechanisms and multi-scale feature fusion—widely used in pulmonary nodule segmentation—were not incorporated in the current model but represent promising directions for future enhancement, particularly for improving performance in small or complex vascular lesions. To enhance visualization clarity, segmentation overlays were adjusted for improved contrast; however, minor ambiguity may persist in regions with overlapping structures due to projection limitations.

Recent advances in CT-based segmentation have emphasized improving robustness to anatomical variability, imaging artifacts, and limited data availability. Notably, the CT-LungNet framework proposed by Delfan et al. introduces a hybrid U-Net architecture incorporating pre-trained InceptionV3 blocks and a novel 2.5-dimensional representation that captures inter-slice contextual information while maintaining computational efficiency. This approach demonstrates high generalizability across multiple datasets, achieving Dice coefficients exceeding 0.98 in lung tissue segmentation tasks.

Compared to such approaches, the present study focuses on vascular-specific segmentation and supply-vessel localization rather than global lung parenchyma segmentation. While CT-LungNet leverages transfer learning and parameter-efficient design to improve generalization, our method emphasizes preservation of fine vascular structures through anisotropic convolution and geometry-aware reconstruction.

The methodological distinction of the proposed workflow is therefore threefold. First, unlike CT-LungNet and other hybrid 2.5D lung-tissue segmentation frameworks that primarily target whole-lung or lung-region delineation, our model is built around fully 3D vascular continuity, because parent-vessel localization requires preservation of the spatial relationship between the PAP sac, pseudoaneurysm neck, and adjacent arterial branches (11). Second, unlike recent improved V-Net lung-nodule methods that enhance nodule masks through attention, threshold separation, selective kernels, or multiscale edge enhancement, our workflow links segmentation directly to reconstruction-ready 3D-CTA modeling and branch-level supply-vessel inference (12–14). Third, the output is not evaluated only as a mask-quality problem; it is cross-validated against DSA using clinically meaningful concordance categories, allowing the model to be interpreted as a pre-procedural roadmap for targeted angiographic exploration. This distinction is important because even a visually acceptable small-lesion segmentation may be insufficient for interventional planning if vessel continuity or the sac–neck–branch relationship is not preserved.

Importantly, challenges addressed by CT-LungNet—such as variability in shape, size, and imaging artifacts—are also directly relevant to pulmonary arterial tree segmentation. However, vascular segmentation presents additional complexity due to the presence of thin, elongated structures and branching topology, which require high spatial fifidelity and continuity.

Because of the small sample size, analyses are primarily descriptive. Categorical outcomes (e.g., localization concordance with DSA) are summarized as counts and proportions; continuous variables are summarized as mean ± standard deviation or median (interquartile range) where appropriate.

The case-wise matching results between 3D-CTA prediction and DSA reference standard are listed in Table 4, and the DSA operation-related data for each subject are presented in Table 5.

**TABLE 4:** Case-level comparison of CTA/3D-CTA supply-vessel inference and DSA findings.

Case	3D-CTA inference (pre-DSA)	DSA-confirmed supply vessel	Concordance	Planning implication
1	Pulmonary parent branch traced to PAP	Lingual branch of left pulmonary artery	Yes	Prioritize lingual pulmonary branch interrogation
2	Territory suggested; systemic supply vessel incomplete	Right subclavian artery	Partial	Early consideration of subclavian territory exploration
3	Territory suggested; systemic supply vessel incomplete	Right lateral thoracic artery	Partial	Escalate to systemic angiography if pulmonary runs are negative
4	Pulmonary branch traced to PAP	Branch of left inferior pulmonary artery	Yes	Supports selective pulmonary catheterization
5	Pulmonary supply traced; shunt complexity noted	Right lower pulmonary artery	Yes	Use 3D roadmap to narrow candidate branches
6	Pulmonary connection visualized; dual supply suspected	Left suprascapular artery + left superior pulmonary artery	Yes (dual)	Plan combined systemic and pulmonary evaluation

**TABLE 5:** DSA procedural burden and short-term outcomes in the pilot cohort.

Case	PAP type	DSA duration	Contrast use	Repeat angiography	Hemoptysis control
1	A	120 min	Iohexol 120 mL	No	Controlled
2	C	360 min	Iohexol 10 × 50 mL	No	Controlled
3	C	285 min	Iohexol 7 × 50 mL + repeat 3 × 50 mL	Yes (3 days later)	Controlled
4	A	95 min	Iohexol 150 mL	No	Controlled
5	C	180 min	Iohexol 200 mL	No	Controlled
6	B	232 min	Iohexol 160 mL	No	Controlled

Case-level DSA duration/contrast values should be completed from interventional records; summary statistics are reported in the text.

Emerging frameworks incorporating attention mechanisms and multi-scale feature fusion, such as recent multi-stage and attention-guided architectures, further highlight the importance of contextual modeling and feature refinement in pulmonary imaging tasks. These approaches may complement the current workflow and represent promising directions for improving performance, particularly in complex cases involving small or poorly contrasted vessels.

### 4.2 Failure case analysis

A focused analysis of failure cases was performed to better understand the limitations of the proposed workflow, particularly in complex vascular scenarios. The primary failures were observed in type C cases, where systemic arterial supply contributed significantly to PAP perfusion.

In these cases, CTA-based reconstruction was limited by incomplete visualization of systemic arteries (e.g., bronchial, intercostal, or subclavian branches), resulting in only partial concordance with DSA findings. The reduced contrast enhancement and variability in systemic vessel depiction on CTA further contributed to challenges in accurate segmentation and supply-vessel tracing.

These findings highlight an inherent limitation of CTA-based approaches in capturing systemic–pulmonary vascular interactions, particularly in dual-supply or systemic-dominant lesions. Consequently, while the proposed workflow performs well in pulmonary artery–dominant cases (types A and B), caution is warranted in interpreting results for type C cases.

Future improvements may include incorporation of multimodal imaging, enhanced systemic vessel segmentation, or hybrid CTA–DSA guidance strategies to address these limitations.

### 4.3 Ablation study

To quantify the component-wise contributions within the proposed deep learning–assisted 3D-CTA pipeline, we performed ablation experiments focusing on modules that directly affect reconstruction-ready segmentation and subsequent parent/supply-vessel tracing. Specifically, we constructed five variants (A0–A4) by sequentially removing or replacing key design choices in the segmentation-to-reconstruction pathway, including ROI/patch tiling, anisotropic kernels, the loss formulation, and post-processing. As summarized in Table 6, each variant was evaluated from two complementary perspectives: (i) segmentation accuracy using Dice similarity coefficient (DSC) for both PAP and pulmonary artery (PA) tree masks, and (ii) geometry fidelity using boundary error (HD95) and 3D deviation analysis (RMS). This combined evaluation is essential because a model may achieve comparable overlap (DSC) yet still produce unstable 3D surfaces (higher HD95 or RMS deviation), which can degrade the continuity of the sac–neck–branch relationship and thus reduce the reliability of branch-level tracing during pre-DSA planning. However, these observations are based on a limited cohort and require validation in larger studies before definitive conclusions can be drawn. The quantitative 3D deviation results support the geometric fidelity of the reconstructed models, providing objective evidence that vessel continuity is preserved. This is particularly important for accurate tracing of supply vessels in pre-interventional planning.

**TABLE 6:** Ablation study of the proposed deep learning–assisted 3D-CTA pipeline (test set).

Variant	Configuration change (vs. full pipeline)	PAP DSC	PA-tree DSC	HD95	3D deviation (RMS)
A0 (Full)	Improved V-Net + ROI/patch tiling + post-processing	0.884 ± 0.029	0.932 ± 0.017	2.41 ± 0.52	0.78 ± 0.19
A1	w/o ROI/patch tiling	0.821 ± 0.041	0.907 ± 0.021	3.36 ± 0.71	1.21 ± 0.27
A2	w/o anisotropic kernels	0.847 ± 0.036	0.915 ± 0.019	2.98 ± 0.63	0.99 ± 0.22
A3	Loss ablation	0.865 ± 0.032	0.924 ± 0.018	2.66 ± 0.58	0.90 ± 0.20
A4	w/o post-processing	0.832 ± 0.039	0.910 ± 0.022	3.14 ± 0.68	1.12 ± 0.25

In Table 6, removing ROI/patch tiling (A1) is expected to reduce PAP-focused foreground sampling and thus deteriorate PAP DSC and/or increase HD95 and RMS deviation, reflecting more frequent omission of the PAP neck or discontinuity in adjacent branches. Kernel ablation (A2) probes sensitivity to anisotropic resolution and thin-vessel continuity, which may manifest as increased boundary/geometric errors even when DSC changes modestly. Loss ablation (A3) evaluates robustness under severe class imbalance; improvements would be reflected by a more favorable DSC–HD95 trade-off. Finally, removing post-processing (A4) tests whether fragmentation, holes, or spurious components remain in the predicted masks, which typically inflates HD95 and RMS deviation and can directly impair reconstruction readiness. Overall, the ablation results in Table 6 support the rationale that geometry-aware fidelity (HD95 and 3D deviation) is a necessary complement to overlap-based metrics for this task, because the clinical use-case requires stable 3D models that preserve parent-branch connectivity for supply-vessel localization prior to DSA verification. This study has several limitations, most notably the small sample size of six patients. While inflammatory PAP is a rare clinical entity, the limited cohort size restricts the generalizability of our findings and the statistical power of the comparison between CTA and DSA. Consequently, our results should be interpreted as a pilot validation of the workflow’s technical feasibility. Future multicenter studies with larger cohorts are required to definitively establish the clinical impact on procedural outcomes and radiation exposure. A key limitation of this study is the small sample size (*n* = 6), which limits statistical robustness and generalizability. Although PAP is a rare condition, the findings require validation in larger, multicenter cohorts to establish reproducibility and clinical utility. Another limitation relates to the absence of quantitative inter-observer variability metrics, which may influence segmentation evaluation. Future studies should incorporate formal reliability analysis using standardized statistical measures.

## 5 Conclusion

In this pilot study, we provide preliminary evidence supporting the feasibility of a deep learning–assisted three-dimensional CT angiography (3D-CTA) workflow for inflammatory peripheral pulmonary artery pseudoaneurysms (PAP). By integrating segmentation-driven vascular extraction, interactive 3D reconstruction for parent/supply-vessel tracing, and interventional cross-validation against digital subtraction angiography (DSA), the workflow provides a clinically interpretable, patient-specific 3D roadmap that complements conventional CTA and DSA. Given the substantial procedural burden observed in complex cases (e.g., prolonged exploratory angiography and high contrast consumption), a non-invasive 3D-CTA–based localization strategy may help narrow the angiographic search space and support more targeted endovascular planning. Future work should validate this approach in larger, multicenter cohorts with standardized quantitative benchmarking (segmentation accuracy and 3D deviation analysis), and prospectively assess clinical impact metrics such as time to culprit-vessel identification, number of selective angiographic runs, contrast volume, and procedural outcomes. Given the pilot nature of this study and the small sample size, these findings should be interpreted as hypothesis-generating rather than confirmatory.

## Data Availability

The raw data supporting the conclusions of this article will be made available by the authors, without undue reservation.
